# 阵发性睡眠性血红蛋白尿症患者C5基因多态性致依库珠单抗治疗无效1例

**DOI:** 10.3760/cma.j.cn121090-20260421-00205

**Published:** 2026-08

**Authors:** 睿卿 周, 薇 周, 艳丽 许, 小卫 陈, 芳琼 李, 顺清 王

**Affiliations:** 1 广州市第一人民医院血液内科，广州 510180 Department of Hematology, Guangzhou First People's Hospital, Guangzhou 510180, China; 2 海思科医药集团股份有限公司，成都 611100 Haisco Pharmaceutical Group Co.,Ltd., Chengdu 611100, China

患者，女，36岁，因“反复头晕、乏力伴贫血18年”入院。患者自2007年（18岁）起无明显诱因反复出现头晕、乏力，多次就诊提示贫血，伴白细胞及血小板轻度降低，间断予补铁治疗，症状未明显改善。2012年因头晕加重就诊，经骨髓穿刺、流式细胞术检测等检查明确诊断为阵发性睡眠性血红蛋白尿症（PNH），予糖皮质激素（具体剂量不详）及间断输血支持治疗。2024年复查外周血，经高灵敏度流式细胞术检测分析PNH克隆示：红细胞99.40％，粒细胞99.70％，单核细胞99.60％。患者于2025年4月开始接受依库珠单抗标准剂量治疗（起始期：前4周每周1次静脉输注600 mg；维持期：第5周静脉输注900 mg，后续每14 ± 2 d静脉输注900 mg），治疗前血常规及生化：HGB 75 g/L，网织红细胞绝对计数（Ret）304.0×10^9^/L，乳酸脱氢酶（LDH）1 893 U/L（正常值范围：120～250 U/L），间接胆红素（IBIL）25.9 µmol/L。治疗4周后复查：HGB 67 g/L，Ret 263.6×10^9^/L，LDH 2 074 U/L，IBIL 24.8 µmol/L，高灵敏度流式细胞术检测分析PNH克隆示：粒细胞99.30％，单核细胞98.71％，Ⅱ型红细胞37.87％，Ⅲ型红细胞60.52％，提示溶血未得到有效控制，贫血无明显改善。鉴于疗效不佳，临床考虑可能存在依库珠单抗原发性耐药，遂行补体C5基因测序分析，结果证实患者存在C5基因杂合型多态性（c.2654G>A），导致编码蛋白第885位精氨酸变为组氨酸（p.Arg885His）。患者经依库珠单抗治疗20周未脱离输血，且LDH高于基线值，明确为原发性耐药。经我院伦理委员会批准（批件号：A-2025-045-01）及患者充分知情并签署知情同意书后，于2025年9月10日停用依库珠单抗，换用口服小分子补体B因子抑制剂（琥珀酸思普可泮）治疗（200 mg/d）。治疗前：HGB 56 g/L，Ret 358.0×10^9^/L，LDH 2 940 U/L，IBIL 34.9 µmol/L，高灵敏度流式细胞术检测分析PNH克隆示：粒细胞99.18％，单核细胞98.96％，Ⅱ型红细胞27.77％，Ⅲ型红细胞47.52％。琥珀酸思普可泮治疗4周后复查，LDH 246 U/L，HGB明显升高。治疗14周后复查，HGB 136 g/L，Ret 167.2×10^9^/L，LDH 247 U/L，IBIL 12.6 µmol/L，高灵敏度流式细胞术检测分析PNH克隆示：粒细胞99.87％，单核细胞99.92％，Ⅱ型红细胞38.61％，Ⅲ型红细胞57.88％；临床症状明显改善。

讨论：本例患者接受标准剂量依库珠单抗治疗4周后，LDH水平仍高达2 074 U/L，HGB无明显改善，流式细胞术检测提示PNH克隆比例未显著下降，随后的基因测序证实患者存在C5基因杂合型多态性（c.2654G>A，p.Arg885His）。在与依库珠单抗相关的原发耐药机制中，C5基因多态性被认为是关键因素之一。本病例因C5基因多态性导致依库珠单抗原发性耐药。对于启动依库珠单抗治疗后，溶血指标如LDH未能快速下降甚至反而升高的PNH患者，应高度怀疑存在此类原发性耐药突变。及时进行C5基因测序是明确诊断的关键。本病例在明确耐药机制后，及时更换为补体B因子抑制剂琥珀酸思普可泮治疗，治疗4周后溶血迅速改善，HGB提升，为C5基因多态性导致的原发耐药患者提供个案依据。C5基因多态性是导致依库珠单抗原发性耐药的重要原因。临床医师应对依库珠单抗治疗后LDH无下降反应的PNH患者保持高度警惕，及时进行基因筛查。一旦确认耐药，采用不同作用机制的补体抑制剂可为患者带来临床获益。

